# Cereal and Juice, Lead and Arsenic, Our Children at Risk: A Call for the FDA to Re-Evaluate the Allowable Limits of Lead and Arsenic That Children May Ingest

**DOI:** 10.3390/ijerph19105788

**Published:** 2022-05-10

**Authors:** Lorenz S. Neuwirth, Ericka Cabañas, Patrick Cadet, Wei Zhu, Morri E. Markowitz

**Affiliations:** 1 SUNY Old Westbury, Department of Psychology, Old Westbury, NY 11568, USA; zhuw@oldwestbury.edu; 2 SUNY Neuroscience Research Institute, Old Westbury, NY 11568, USA; ecabanas@oldwestbury.edu (E.C.); cadetp@oldwestbury.edu (P.C.); 3 SUNY Old Westbury, Department of Biology, Old Westbury, NY 11568, USA; 4 Lead Poisoning Treatment and Prevention Program, Montefiore Medical Center, Bronx, NY 10467, USA; mmarkowi@montefiore.org

**Keywords:** lead poisoning (Pb), arsenic poisoning (As), rice cereal, juice, Federal Drug Administration (FDA), infants, toddlers, children, manufacturing safety guidelines, risk factors

## Abstract

Eliminating heavy metal contamination of foods is a goal yet to be achieved in the U.S. In recent months, efforts have been underway to have the Food and Drug Administration (FDA) re-evaluate the permissible limits of lead (Pb) and arsenic (As) allowable in cereals and juices aimed for consumption by children. This report discusses the recent scientific literature that support proposed revisions in these limits. It presents proactive suggestions for the FDA to consider in its response to concerns of ongoing Pb and As exposures in food and drinks. While more scientific studies are needed to better define ‘safe’ levels of Pb and As exposures and ingestion of these elements in general are neurotoxic, the higher sensitivity of children to these toxic elements makes it imperative that the FDA adjust standards to be most protective of infants, toddlers, and children.

## 1. Introduction

Since 2013, the magazine Consumer Reports [1,2] has indicated an emerging concern over the lead (Pb) and arsenic (As) content in foods and drinks that exceed the levels permissible in drinking water. The pediatric population consumes these neurotoxic foods and this poses risks to their developing central nervous systems (CNS), even at low exposure levels. A recent investigation of 105 infant cereal samples found As in infant rice cereals to be eightfold higher, up to 85 parts per billion (ppb), than the US Environmental Protection Agency’s (EPA) [3] legal limit of 10 ppb of As in drinking water [4]. Notably, As was found in baby rice cereals and teething biscuits [5]. Another study, by the “*Clean Label Project*” (CLP), tested 500 baby food products consisting of 86 infant formulas, 30 baby cereals, 105 baby food jars, 138 baby food pouches, 36 toddler juices and drinks, and 138 toddler snacks from 60 brands that are currently being distributed and sold [6]. The investigators found that 65% of the baby foods had detectable levels of As, 36% had detectable levels of Pb, and 58% had detectable levels of cadmium [6]. An analysis of data collected by the US Federal Drug Administrations (FDA) in its study, “*Total Diet Study data—On Toxic and Nutritional Elements Summaries of Multi-Year Results from 2006–2013*”, on 2164 baby food samples, identified Pb in 20% of the samples (i.e., a one in five risk factor) [7]. More specifically, fruit juices were more likely to have Pb in the samples: 89% of grape juices, 67% of mixed fruit juices, 55% of apple juices, and 45% of pear juices, attesting to the differential risks based upon the type of food ingested [7].

The issue of contaminated foods is not limited to the US. Recent reports have shown that As has been found in cereal and other foods in Belgium [8], Argentina [9,10], Korea [11], and Spain [12]. Prior concerns regarding As exposure were raised at the global level as they pertained to infant rice cereal [13]. Further, in Taiwan, consumption of As has been reported to alter both metabolism and DNA methylation and was associated with neurodevelopmental delays in children [14]. Thus, As-containing rice crops, from which most cereals are manufactured, may require more scrutiny than what was and currently is considered acceptable. Further, the As-exposure limits (i.e., both nationally and internationally), given the context of the increasing amounts identified in infant, toddler, and children cereals, should themselves raise the level of concern for more rigorous or modified oversight of the production of these food and drink products globally.

It is important to note that both organic and non-organic foods may be unsafe. Recently, there has been an increase in organic chicken egg farming within urban areas (e.g., Denver, Los Angeles, Miami, and New York) of the US. These urban farmers raise chickens to lay eggs with the belief that they are nutritious food sources that occur naturally [15]. However, Leibler et al. [16] found that, of 201 eggs collected from urban farmers in the greater Boston Massachusetts area, 98% were Pb-contaminated (*M* = 0.10 μg/dL, *SD* = 0.18). Moreover, they estimated that children consuming these eggs would increase their blood lead levels (BLL) by 0.9–1.5 μg/dL [16].

These studies suggest that toxic element exposures persist through a range of foods likely to be ingested by infants and children, posing risks for neurotoxicant exposures. Currently, systematic screening of young children for toxic element exposure by testing biological specimens is only performed for Pb; and even then, it is only mandated at ages 1 and 2 years and in a handful of states within the US [17]. Yet early exposure in life to toxic elements can directly influence the growth of children and can negatively influence their neurodevelopment, which are risk factors for being diagnosed with developmental disabilities later in life. It is imperative that the FDA reliably monitors and tests for toxic element exposures in US foods and drinks, with limits set based upon scientific studies relating ingestion to health outcomes (i.e., an I:HO ratio or index for infants, toddlers, and children, perhaps) for both Pb and As. In addition to the FDA, primary care providers are essential parties in helping parents understand how to limit the risks of dietary toxic element exposure in their children through educational outreach efforts and consistently implmented biomarker screening.

### 1.1. Gastrointestinal Absorption in Children

Calcium (Ca) and Pb compete with one another in physiological systems. In cells, Pb may enter via the Ca channels present in the cell membrane [18]. In the gastrointestinal (GI) tract, Ca absorption occurs by two mechanisms: (1) transcellularly, i.e., through cells, which likely allows Pb cell entry, and (2) paracellularly, i.e., between cells, which may also accommodate Pb entry (*For Review See* Bronner [19]). Children and young animals have developmentally mature GI systems, but their absorption kinetics are markedly different from that of mature humans and animals [20]. Alexander [21] and Ziegler et al. [22] reported 40–50% Pb absorption in children vs. 10–15% in adults. However, these estimates may not be applicable to all situations of Pb ingestion. Most Pb compounds are poorly soluble in water at pH 7, whereas more are released in acid [23]. This implies that Pb ingested in a solid-like baby food has a different bioavailability than Pb ingested already dissolved in a liquid such as juice [23]. During times when children are between meals and/or fasting, they are at increased risk for Pb absorption from the GI tract since competition for absorption pathways is lacking; during these specific time-periods, they also require higher demands in gut metabolism [24,25].

Both iron and Ca status affect Pb absorption and retention [26]. In animal studies, increasing Ca intake is a nutritional intervention to counteract against Pb exposure. In Pb-exposed children, dietary Ca intakes are inversely associated with their BLLs [26,27,28]. This can be attributed to competition between Pb and Ca for Ca-channel-mediated entry [29]. However, additional Ca intake above the recommended daily intake has little effect on changing BLLs over time [27,28]. Furthermore, children with prior Pb ingestion resulting in bone Pb accumulation (i.e., the site with the most Pb in the body with chronic exposure), are at increased risk for accelerated bone Pb release into the blood when deficient in Ca-intake [24], with subsequent renewed (neuro)toxicity [25,29,30].

Age is a critical risk factor in Pb poisoning; the prevalence is higher in children, with greatest concern for children between 18 and 30 months of age [28]. This important distinction between children and adults is attributable to normative nonnutritive oral behavior in young children/infants as well as to the efficiency of Pb-absorption. Thus, Pb toxicity, especially its effects on brain development/function, appears to be age-dependent, with greater potential effects on cognitive and behavioral outcomes noted after prenatal and early childhood poisonings [31,32,33].

### 1.2. Limiting Lead Exposure from Foods

Given this difference in bioavailability of ingested Pb, it follows that allowable exposure limits should be lower for children than adults. Currently, the FDA and the US Center for Disease Control and Prevention (CDC) recommend an Interim Reference Level of 3 μg/day (i.e., ingestion) for children and 12.5 μg/day for adults [34]. However, how does the public know how much Pb exists in any food and drink source they purchase commercially and subsequently consume? Commercial food and drink products have labels indicating the caloric and nutrient content per serving. These are intended to inform the public of what they are consuming so that people can make conscious, health-based choices. About one-third of consumers read all or part of the label, implying their concern about the health impact of the foods and drinks they consume [35]. However, comparable information about neurotoxicants such as Pb are not indicated on food and drink packaging. Arguably, this situation should be re-evaluated given children′s higher absorption rates and sensitivity to toxic elements such as Pb.

### 1.3. Childhood Lead and Arsenic Poisoning and Future Intellectual and Behavioral Problems

Dakeishi et al. [36] reported on the neurotoxic and lethal impacts of As poisoning that occurred in Japanese infants in 1955. The source was contaminated milk powder, leading to the ingestion of more than 500 μg/kg/day. More than 100 infants died. Clinically evident poisoning was calculated to occur after ingesting approximately 60 mg of As. Follow-up examination of the infants at 50-years of age revealed intellectual disabilities, neurological diseases, and other disabilities [36]. Vhater [37] further reviewed the literature on As and reported that inorganic and methylated As crosses the placenta in human clinical and animal experimental studies, thus providing noteworthy evidence of increased risk for fetal exposure, teratogenic effects, and developmental neuropathies. Delayed effects of intrauterine As-exposure have included increased mortality due to lung disease in young adults, possibly as the result of early epigenetic modifications [37]. Moreover, reports have begun to shed light on indirect As/Ca relationships that may increase and stabilize the bioavailability of As in aqueous solutions [38,39,40], which may, in turn, have effects on Ca-signaling pathways [41]. The results are a unique chronic exposure profile that may remain in a dormant form (i.e., asymptomatic) for many years while still posing a neurotoxic risk. It is important to note that As chemistry is complex, with toxicity being dependent upon the specific form to which one is exposed, thus making the identification of As absorption pathways more elusive than that reported for Pb. As a result of these challenges, As toxicity remains problematic.

Interventions to decrease As toxicity have employed the use of essential elements. For example, studies of rodent brains have shown that As poisoning alters apoptotic caspases and antioxidant-related enzymes, resulting in oxidative stress [42]. Administration of Ca, selenium, and magnesium protect against these As-induced oxidative stress effects [43]. Similarly, Ca and zinc supplementation have been shown to protect against Pb-induced oxidative stress due to altered antioxidant enzymes and lipid peroxidation in the developing mouse brain [44]. Taken together, there may be both divergent and convergent downstream mechanisms of action that As and Pb may share through the oxidative stress pathways, which are susceptible to treatment interventions.

### 1.4. Food and Juice Concerns in Modern Times

Independent nonprofit organizations have clearly demonstrated levels of toxic element contamination in foods and drinks intended for consumption by infants, toddlers, and children that exceed standards set by: (1) the World Health Organization (WHO), (2) the FDA, and (3) the State of California Proposition 65 for daily Pb consumption [45]. The alarming results of these studies raise the following issues and concerns about possible federal agencies’ responses: (1) the government may claim that its current monitoring systems are sufficient and definitive, (2) the government may suggest that the toxic element exposure levels described by these nonprofit, nongovernmental organizations are not of public health concern, (3) the government may simply dismiss these efforts of nonprofit organizations to raise awareness of the potential ongoing exposure to these neurotoxic elements as irrelevant, (4) which may, in turn, mislead to the government to reallocate funds for ongoing monitoring to other programs, and (5) if the government does not adequately monitor Pb- and As-exposures and neurotoxicity occurs in infants, toddlers, and children, then it may create a sense of public distrust of government and business corporations that manufacture foods and drinks that target the next and future generations of children.

## 2. Conclusions

While the Joint Food and Agriculture Organization of the United Nations/WHO Expert Committee on Food Additives [46] indicated that, for Pb, there is no safe exposure level, the FDA [34] still has not adjusted its guidelines with respect to food sources of exposure. The persistent negative impacts that Pb-poisoning from any exposure throughout the life cycle has on the economy are well-established [47,48,49]. It is the government’s responsibility to protect the public’s health by having effective safety regulations and also ensuring that they are in place and being adhered to. To a resurgent awareness of Pb-exposure from old (e.g., water supplies) and new (e.g., fracking) sources [31], as an ever-growing health conscientious people, we as an informed and conscientious people must now renew concerns about food contamination reminiscent of the American journalist/novelist Upton Sinclair’s 1906 novel “*The Jungle*”. Foods and drinks with product sales directed towards infants, toddlers, and children require more stringent regulations based on solid scientific study and full public disclosure, with such stringent measures also applicable to advertising tactics.

**Figure 1:**
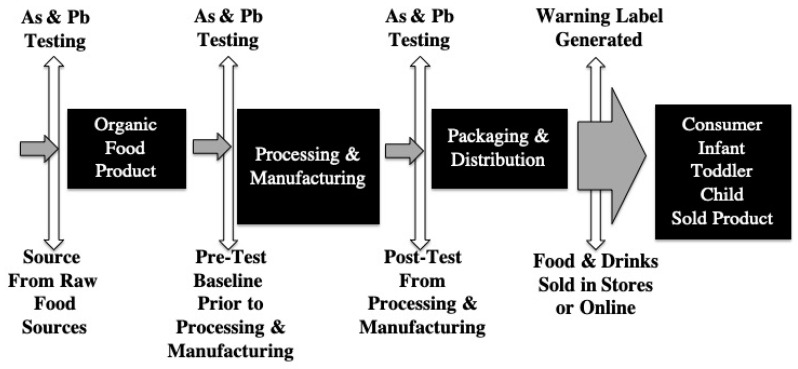
A schematic suggesting how to achieve As- and Pb-free food by sequential testing throughout the manufacturing process. This process aims to limit human As- and Pb-exposures, especially for infants, toddlers, and children. The grey directional arrows indicate the source-to-consumer process; the white double arrows indicate the testing phase for assuring As- and Pb-free foods; and the black boxes indicate the steps in the manufacturing process for achieving the production of As- and Pb-free foods from farm-to-table. The end goal is to establish clear limits for As and Pb in foods and drinks, and to inform the consumer through warning labels for all consumable goods sold in stores or online.

This raises the civic need to have a stronger set of checks and balances beyond that of the FDA’s “*Arsenic in rice and rice products*” [50] and “*Total Diet Study*” [51]. That begins with government regulations based on up-to-date knowledge of toxic element effects, especially those in children due to an increased risk of neurotoxicity. It includes acknowledging that food producers and sellers share responsibility for the safety of their products, especially when their products target children as their consumer market. A schematic diagram for a potential model to best address this issue when developing infant, toddler, and children’s food and drink products is illustrated in Figure 1. Currently, similar public concerns are being addressed in Nigeria [52], Spain [12], and France [53]. Yet, in the US, recent reports in the *Advances in Pediatrics regarding Lead Poisoning in Children* [54] failed to mention infant, toddler, and children’s food and drink Pb and As contamination as potential sources of toxic element exposure. Furthermore, recent international reference manuals and guides on food safety (“*Food Safety Aspects of Grain and Cereal Product Quality*” [55] and “*Safety of Food and Beverages: Cereals and Derived Products*” [56]) restrict their focus to microbial growth; they fail to mention any concerns regarding neurotoxicants such as Pb or As. This can and must be rectified. The modern technology for determining the contents of toxic elements in foods such as rice exists [57].

One way to diminish neurotoxic element exposures would be to add updated intake limits to each food’s product label, just as is currently required for nutrients. This would be no different from the legal requirement for other risks, such as the label stating: “*U.S. Surgeon General Warning: Smoking Causes Lung, Cancer, Heart Disease, Emphysema, And May Complicate Pregnancy*” that appears on tobacco products. In the present case, it could be presented in the same manner for labels on foods and drinks, e.g., “*U.S. Surgeon General Warning: Product May Contain Lead or Arsenic*” (followed by the amount and compared to the federal/government standard). Such a transparent and informed approach, by re-evaluating the allowable Pb or As limits for commercially sold food and drink products and separating these allowable limits between infants, toddlers, children and adults (i.e., establishing clear daily limits for the range of developmental time-periods), may lessen the social and economic costs associated with childhood Pb- and As-poisoning across the lifespan [17]. The goal should allow consumers to be made aware of the toxic elements in their foods with the use of warning labels so that informed health choices can be made and the growth and neurodevelopment of infants, toddlers, and children left unaffected by Pb- and As-exposures. These warning labels should be formalized across all food and drink products for quality assurance, safety, transparency, and establishing quality-sourced consumable goods for people of all ages to consume safely.

## Data Availability

Not applicable.

## Abbreviations

Lead poisoning (Pb); arsenic poisoning (As); parts per billion (ppb); Environmental Protection Agency (EPA); Clean Label Project^TM^ (CLP); Environmental Defense Fund (EDF); Federal Drug Administration (FDA); blood lead levels (BLLs); calcium (Ca); gastrointestinal (GI); Center for Disease Control and Prevention (CDC); World Health Organization (WHO).
